# Interactive effects of the low‐carbohydrate diet score and genetic risk score on Hypo‐HDL‐cholesterolemia among Korean adults: A cross‐sectional analysis from the Ansan and Ansung Study of the Korean Genome and Epidemiology Study

**DOI:** 10.1002/fsn3.2909

**Published:** 2022-04-29

**Authors:** SoHyun Park, Min‐Jae Jang, Min Young Park, Jun‐Mo Kim, Sangah Shin

**Affiliations:** ^1^ Department of Food and Nutrition Chung‐Ang University Gyeonggi‐do Korea; ^2^ Department of Animal Science and Technology Chung‐Ang University Gyeonggi‐do Korea; ^3^ Department of Molecular Pathobiology NYU College of Dentistry New York New York USA

**Keywords:** Ansan Ansung cohort, dietary factor, genetic risk score, hypo‐HDL‐cholesterolemia, low‐carbohydrate diet score

## Abstract

This cross‐sectional study investigated the interaction between the genetic risk score (GRS) and abnormal high‐density lipoprotein (HDL) cholesterol lipid levels, which are modified by low‐carbohydrate diets (LCDs) and their effects on the prevalence of hypo‐HDL‐cholesterolemia (hypo‐HDL‐C) in Korean adults. Baseline data were obtained from the Ansan and Ansung study of the Korean Genome and Epidemiology Study (KoGES), conducted from 2001 to 2002, that targeted 8,314 Korean adults aged 40–69 years, including old men (47.6%) and women (52.4%), and whole genomic single nucleotide polymorphism (SNP) genotyping was performed. We identified 18 SNPs significantly associated with hypo‐HDL‐C in the proximity of several genes, including *LPL*, *APOA5*, *LIPC*, and *CETP*, and calculated the GRS. The low‐carbohydrate diet score (LCDS) was calculated on the basis of energy intake information from food frequency questionnaires. Furthermore, we performed multivariable‐adjusted logistic modeling to examine the odds ratio (OR) for hypo‐HDL‐C across tertiles of LCDS and GRS, adjusted for several covariates. Among participants in the highest GRS tertile, those in the highest tertile of the LCDS had a significantly lower risk of hypo‐HDL‐C (OR: 0.759, 95% CI (confidence interval): 0.625–0.923) than those in the lowest tertile of the LCDS. In the joint effect model, the group with the lowest GRS and highest LCDS was found to have the lowest risk of hypo‐HDL‐C prevalence. This study suggests that individuals with a high genetic risk for low HDL concentrations may have a beneficial effect on a lower intake of carbohydrates.

## Graphical abstract

This study investigated the interaction between the genetic risk score (GRS) and abnormal blood lipid levels, which are modified by low‐carbohydrate diets (LCDs) and their effects on the prevalence of hypo‐HDL‐cholesterolemia (hypo‐HDL‐C) in Korean adults. The highest prevalence risk was observed in individuals with low LCD and high. In addition, at any level of GRS, a higher LCD decreased the hypo‐HDL‐C prevalence, and at any level of LCD, a higher GRS increased the hypo‐HDL‐C prevalence. An LCD could be effective in reducing hypo‐HDL‐C in individuals with a high GRS for low HDL levels among Korean adults.

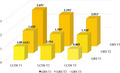

## 1 INTRODUCTION

The prevalence of cardiovascular diseases (CVDs), a leading cause of death worldwide, is increasing in many parts of the world, including Korea (Virani et al., 2020). Dyslipidemia, defined by increased levels of total cholesterol, triglycerides (TGs), low‐density lipoproteins (LDLs), and decreased levels of high‐density lipoprotein (HDL) cholesterol, is a well‐known independent risk factor for CVDs (Kim & Oh, 2013). High‐density lipoprotein cholesterol levels significantly affect CVD, despite the lack of significant association between CVD and total cholesterol levels (de Freitas et al., 2011). HDL cholesterol lowers the risk of CVD (Farmer & Liao, 2011; Florentin et al., 2008) and reduces excess cholesterol from atheromas and cells (Hewing et al., 2012).

Hypo‐HDL‐cholesterolemia (hypo‐HDL‐C) and hyper‐triglyceridemia are major risk factors of dyslipidemia in the Korean population (Frank et al., 2014). The prevalence of hypo‐HDL‐C is high and has gradually increased among Korean populations (Kim et al., 2006). Decreasing HDL cholesterol levels in Korea are associated with westernized diets and decreased physical exercise (Choi et al., 2011). Although obesity in Asian populations is less prevalent than in Western populations, HDL cholesterol levels were lower in the Korean population than in Western populations, suggesting that Asian and Western populations have genetic differences affecting the regulation of HDL cholesterol levels (Kim et al., 2011; Moon et al., 2015).

Hypo‐HDL‐C is determined by genetic and dietary factors, weight changes, obesity, and lifestyle factors such as alcohol consumption, smoking status, and physical activity (Kim et al., 2006), among which unhealthy diet constitutes a major risk factor. The Korean population, which consumes potato, legumes, vegetables, mushrooms, fish, seafood, and seaweeds, which comprise a low‐carbohydrate diet, has a low risk of hypo‐HDL‐C (Lee & Kim, 2018). A high dietary carbohydrate‐to‐fat ratio increases the risk of hypo‐HDL‐C in women (Lee & An, 2020). In contrast, low‐carbohydrate intake decreases the risk of hypo‐HDL‐C among Korean adults (Kim et al., 2019).

Other major risk factors for hypo‐HDL‐C are genetic. A study conducted in Roma and Hungarian populations showed that six single nucleotide polymorphisms (SNPs) in lipase C and hepatic type (*LIPC*) and five SNPs in cholesterol ester transfer protein (*CETP*) were significantly associated with an increasing TG/HDL cholesterol ratio, which raises the risk of CVD (Piko et al., 2020). SNP *rs6564851* in beta‐carotene oxygenase 1 (*BCMO1*) is positively associated with HDL cholesterol levels among the US population (Clifford et al., 2013). However, most previous studies have investigated only single SNPs to reveal the factors associated with HDL cholesterol levels, and more studies on the association of whole genetic effects with HDL cholesterol levels are required.

To investigate the associations between HDL cholesterol and diseases, studies investigating interactions between genetic factors and diet are necessary. A study conducted in Framingham reported that a high polyunsaturated fatty acid (PUFA) intake is more significantly associated with an increased HDL cholesterol concentration in women having the apolipoprotein A5 (APOA1) G‐A polymorphism than in those having the G‐G polymorphism (Ordovas et al., 2002). Furthermore, the interaction between dietary intake of n‐3 and n‐6 PUFA and fatty acid desaturase 1 (*FADS1*) genetic polymorphisms may play a role in modulating plasma cholesterol concentrations (Lu et al., 2010).

Most studies focus on the interaction of a single causative SNP with a dietary factor in hypo‐HDL‐C. The genetic risk score (GRS) is a useful measurement to evaluate the effect of multiple associated loci of interest (Iwata et al., 2012; Peterson et al., 2011). A study on the Southeast Asian population showed a significant correlation of GRS with waist circumference and TG levels influenced by low‐protein intake (Alsulami et al., 2020).

The interactive effects between dietary and genetic factors on hypo‐HDL‐C among the Korean population have not been previously investigated using a genome‐wide association study (GWAS). Hence, we investigated the interactive effects of low‐carbohydrate diet score (LCDS) and GRS on hypo‐HDL‐C in Korean adults based on data from the Ansan and Ansung study, which could facilitate personalized nutrition.

## 2 PARTICIPANTS AND METHODS

### 2.1 Study population

Baseline data were obtained from the Ansan and Ansung study of the Korean Genome and Epidemiology Study (KoGES) conducted from 2001 to 2002 among 8314 Korean adults aged 40–69 years including men (47.6%) and women (52.4%), and whole genomic SNP genotyping was performed. This study was performed to investigate the environmental and genetic causes of common chronic diseases, such as metabolic diseases and CVDs, in South Koreans (Kim, Han et al., 2017).

**FIGURE 1:**
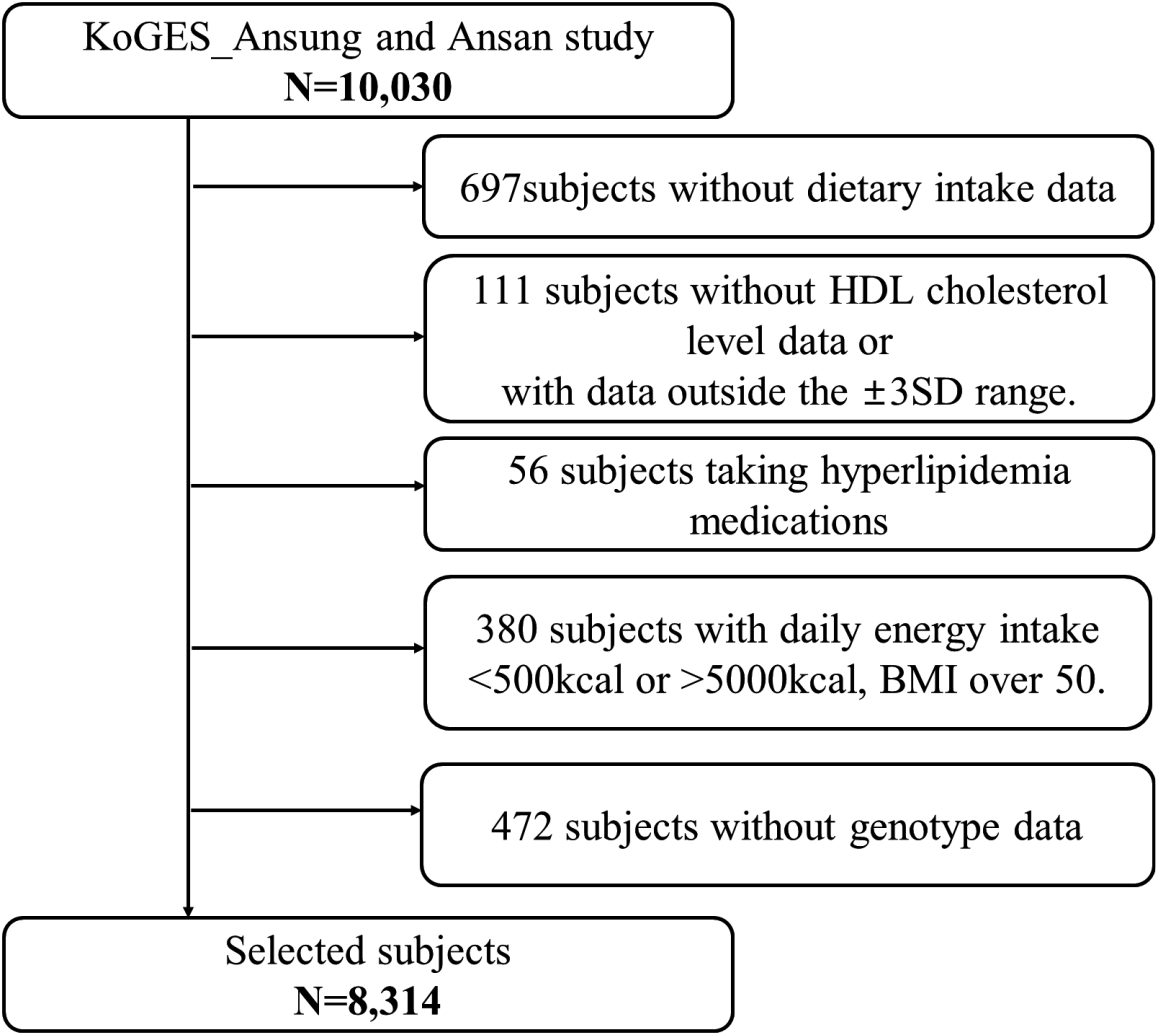
Flowchart of the study participants

From the 10,030 subjects initially included in the Ansan and Ansung study, we excluded subjects without dietary intake data (*n* = 697), HDL cholesterol data, and those with data outside the 3SD range from the mean value (*n* = 111). We excluded participants taking hyperlipidemia medications (*n* = 56), participants with an inadequate range of energy intake (<500 kcal or >5000 kcal), those with a body mass index (BMI) range over 50 kg/m^2^ (*n* = 380), and those with missing genotype data (*n* = 472). In addition, all participants with a history of hyperlipidemia were excluded. Thus, 8314 participants were included (Figure 1). The protocol for the use of data was obtained from the KoGES (4851–302) and approved by the National Research Institute of Health, Centers for Disease Control and Prevention, Ministry for Health and Welfare, Republic of Korea.

### 2.2 Definition of hypo‐HDL‐C

High‐density lipoprotein cholesterol levels were measured in blood samples collected after 8–14 h of overnight fasting. Participants with HDL cholesterol levels below 40 mg/dl were diagnosed with hypo‐HDL‐C based on the Korean Society of Lipid and Atherosclerosis guidelines (Cohen et al., 2004).

### 2.3 Low‐carbohydrate diet score (LCDS) measurement

Total energy and macronutrient intake data were collected using 106 food items from valid semiquantitative food frequency questionnaires developed for KoGES and were validated in a previous study (Ahn et al., 2007). According to the 2020 Dietary Reference Intakes for Korean (KDRIs) by The Ministry of Health and Welfare Korean Nutrition Society, the recommended intake of carbohydrates for the Korean population is 55%–65% of the total energy intake.

For the LCDS, carbohydrate, protein, and fat intake was calculated using standard conversion factors as a percentage of energy (4 kcal/g for carbohydrate and protein, 9 kcal/g for fat). Participants were given 0 to 10 points for each three nutrient intake levels by sex. The sum of the points represented the LCDS, which ranged from 0 to 30 points. A high or low LCDS indicates a lower‐ or higher‐carbohydrate diet, respectively, specific to this study population (Table 1). The LCDSs were divided according to sex and age into tertiles (T1, T2, and T3 for the lowest, middle, and highest LCDS group, respectively) for further analysis.

**TABLE 1:** Characteristics according to the LCDS tertiles among Korean adults

	LCDS T1	LCDS T2	LCDS T3	*p*‐value^a^
*N*	2749	2791	2774	
Men (%)	1,307 (47.5)	1,324 (47.4)	1,324 (47.7)	.9762
Age (year)	52.1 ± 8.9	52.2 ± 8.8	52.0 ± 8.9	.7541
BMI (kg/m^2^)	24.5 ± 3.2	24.7 ± 3.0	24.7 ± 3.1	.0098
HDL‐cholesterol (mg/dl)	43.8 ± 9.2	44.2 ± 9.1	45.1 ± 9.5	<.0001
Household income				
<1,000,000 KRW	1,206 (44.7)	906 (32.8)	715 (26.1)	<.0001
<3,000,000 KRW	1,189 (44.1)	1,343 (48.6)	1,371 (50.0)	
≥3,000,000 KRW	302 (11.2)	514 (18.6)	654 (23.9)	
Drinking status				
Alcohol drinker	1,589 (58.0)	1,451 (52.1)	1,321 (47.8)	<.0001
Nondrinker	1,149 (42.0)	1,333 (47.9)	1,440 (52.2)	
Smoking status				
Current smoker	693 (25.5)	693 (25.0)	709 (25.8)	.5806
Past smoker	407 (15.0)	693 (25.0)	709 (25.8)	
Nonsmoker	1,620 (59.6)	1,643 (59.2)	1,588 (57.8)	
Physical activity				
None	1,453 (54.4)	1,356 (49.3)	1,270 (46.5)	<.0001
<1 h	619 (23.2)	768 (27.9)	700 (25.6)	
<2 h	267 (10.0)	335 (12.2)	444 (16.3)	
≥2 h	332 (12.4)	291 (10.6)	316 (11.6)	
Nutrient intake				
Energy (kcal)	1,775.4 ± 593.1	1,933.2 ± 566.8	2,099.5 ± 639.7	<.0001
Carbohydrate (g)	344.0 ± 116.5	345.9 ± 101.0	334.5 ± 98.5	.0008
%E of carbohydrate	78.8 ± 3.7	72.4 ± 3.3	64.6 ± 5.3	<.0001
Protein (g)	50.5 ± 17.8	64.1 ± 19.3	82.7 ± 28.7	<.0001
%E of protein	11.5 ± 1.2	13.4 ± 1.2	15.8 ± 1.9	<.0001
Fat (g)	19.2 ± 9.1	30.6 ± 12.5	46.1 ± 20.1	<.0001
%E of fat	9.7 ± 2.9	14.1 ± 3.0	19.6 ± 4.3	<.0001

Note

Values are shown as *N* (%) and mean ± *SD*.

Abbreviations: %E, percent energy; KoGES, Korean Genome and Epidemiology Study; KRW, Korean won (1 million KRW is approximately 860 USD); LCDS, low‐carbohydrate diet score; T, tertile.

^a^ *p*‐values were obtained from the generalized linear model for continuous variables, and the chi‐square test for categorical variables.

### 2.4 GRS estimation

Genomic DNA was extracted from peripheral leukocytes collected from study participants (Cho et al., 2009). Genotyping was performed using the Affymetrix Genome‐Wide Human SNP array 5.0 (Affymetrix, Inc.). Available SNPs were filtered for call rate, minor allele frequency (MAF), and Hardy–Weinberg equilibrium by referring to the relevant criteria (Cho et al., 2009). SNP imputation was conducted using the IMPUTE program (Marchini et al., 2007). The imputation was based on NCBI build 36 and dbSNP build 126 and used HapMap data from 90 individuals from Tokyo, Japan, and Han Chinese in Beijing, China, founders in HapMap, as a reference (HapMap release 22). After removing SNPs with MAF < 0.01 and SNP missing rate >0.05, 1.8 million imputed SNPs were collected for association analyses with the hypo‐HDL‐C trait (Cho et al., 2009).

A GWAS between SNPs and HDL cholesterol levels was performed using PLINK version 1.09 (https://www.cog‐genomics.org/plink2) tested by the linear model after adjustment for formulation and statistical model description (Purcell et al., 2007). Linear association analysis was performed using 1,590,162 SNPs. The threshold criterion was set to −log_10_ *p*‐value >6 for 6K SNPs from GWAS (Kato et al., 2011; Kim, Kim et al., 2017; Kim, Han et al., 2017). Linkage disequilibrium (LD) clumping and LD analysis were performed using the causal variants identification in associated regions (CAVIAR) program to identify causal SNPs located in trait‐associated regions (Hormozdiari et al., 2014). SNP locations and nomenclature were defined by Ensembl. The GRS was calculated for each subject with the 18 most strongly associated SNPs according to the following model (1):

(1) $$Y_{\mathrm{ijk}}=\mu +G_{i}+\ldots e$$

$$\text{GRS}=\sum_{i=1}^{n}\text{risk allele in}{\text{SNP}}_{i}\times {\text{weight}}_{i}$$

where *Y*_ijk_ is the observed value of the hypo‐HDL‐C trait, μ is the mean of the samples, *β*_1_(age_i_) is the covariate of age (level: 40–69), *β*_2_(sex_i_) is the covariate of sex, SNP_k_ is the effect of SNP, and e_ijk_ is the random error. The beta values represent the effective size of increasing the HDL cholesterol level in each SNP. Therefore, a high or low GRS is associated with a high risk or low risk of having low HDL cholesterol levels, respectively. For the analysis, GRS was divided into tertiles, T1, T2, and T3, for the lowest, middle, and highest GRS groups, respectively.

### 2.5 Covariates

The general characteristics of this study population were collected using questionnaires and anthropometric and clinical measurements including height, weight, and BMI (weight [kg] ÷ height^2^ [m^2^]). Sociodemographic data were categorized based on two cities of residence (Ansan and Ansung), household income level (by monthly Korean won [KRW] (1 million KRW is approximately 860 USD); <1 million, <3 million, and >3 million), daily physical activity (none, <30, 30–60, 60–90, 90 min to 2, 2–3, 3–4, 4–5, or >5 h), drinking habits (drinker and nondrinker), and smoking habits (current, past, or nonsmoker). To determine the drinking status, data were collected using the question, “do you drink or not for several reasons (regional reason, etc.)?,” and smoking status was determined based on WHO criteria, “Have you smoked more than 100 cigarettes?,” and “Do you still smoke?”

### 2.6 Statistical analysis

Statistical analyses were performed using the SAS software (version 9.4; SAS Institute, Inc.), and a *p*‐value < .05 was considered statistically significant. Categorical data are presented as the number of subjects (%) and continuous data as the means with standard deviation. Multivariable‐adjusted logistic regression analysis and joint interaction analysis were performed to examine the OR and 95% CI for hypo‐HDL‐C across the tertiles of each LCDS and GRS, adjusting for covariates. To analyze the *p*‐values for the trends, regression modeling was performed.

Model 1 was adjusted for sex (men and women) and age (continuous). Model 2 was adjusted for sex (men and women), age (continuous), BMI (continuous), energy intake (continuous), household income level (<1 million, <3 million, and >3 million), alcohol drinking status (drinker and nondrinker), smoking status (current, previous, or nonsmoker), physical activity (none, <30, 30–60, 60–90, 90 min to 2, 2–3, 3–4, 4–5, or >5 h), and residence (Ansan and Ansung). In addition, association trends were calculated by modeling the tertile medians of each LCDS and GRS as continuous variables and deriving the *p*‐value using the Wald test.

## 3 RESULTS

### 3.1 Characteristics of the study population

General participant characteristics based on LCDS tertiles are described in Table 1. Subjects categorized in LCDS T3 tended to have higher BMI (*p* = .0098) and house income level (*p* < .0001), fewer subjects were current drinkers (*p* < .0001), and more were physically active than those in LCDS T1.

The nutrient intake of subjects across the LCDS tertiles is shown in Table 1 and Table S1 (Supporting Information). Intakes and energy percentage from protein and fat were significantly lower in LCDS T1 than in LCDS T3 (*p* < .0001). Intakes and energy percentage from protein and fat were significantly higher in LCDS T1 than in LCDS T3 (all *p* < .0001). Intakes of other nutrients increased as LCDS increased (all *p* < .0001) (Table S1).

### 3.2 Hypo‐HDL‐C GWAS

A set of 1,590,162 SNPs was applied to GWAS (Table 2). The greatest and lowest numbers of SNPs were located on human chromosome 2 and 19, respectively. The average interval of the available SNPs located by chromosome was 1,856.0 kb; chromosome 19 and 6 had the widest and narrowest interval, respectively.

**TABLE 2:** Identified SNPs and average interval distances between adjacent SNPs in the 22 human autosomes among Korean adults

Chromosome	Number of SNPs	Average interval (kb)	Standard deviation (kb)	Total distance (bp)
1	124,121	1,985.1	6,0894.7	246,391,884
2	142,070	1,708.2	11,386.2	242,679,861
3	111,287	1,790.7	14,436.3	199,285,064
4	101,257	1,887.4	12,004.2	191,109,993
5	108,867	1,658.6	11,660.6	180,569,964
6	120,023	1,421.9	9,528.9	170,659,819
7	88,026	1,802.5	13,295.7	158,663,166
8	95,470	1,530.5	12,331.7	146,112,942
9	77,035	1,816.8	94,118.5	139,952,233
10	89,719	1,506.9	11,001.6	135,200,383
11	84,757	1,584.0	11,582.3	134,256,672
12	77,661	1,702.7	6560.0	132,229,515
13	65,544	1,465.1	3,539.7	96,027,786
14	51,388	1,693.0	4,329.3	86,998,491
15	42,947	1,903.9	8,552.0	81,763,030
16	39,351	2,253.2	52,705.0	88,662,122
17	30,603	2,568.0	8,240.5	78,586,573
18	46,858	1,621.2	8,674.0	75,966,267
19	17,914	3,549.4	61,097.3	63,580,903
20	36,213	1,721.7	12,417.9	62,344,784
21	21,109	1,747.9	22,825.8	36,895,145
22	17,942	1,912.9	6,455.9	34,319,536
Total	1,590,162			2,782,256,133
Average		1,856.0	2,0801.7	

Abbreviation: SNP, single nucleotide polymorphism.

**FIGURE 2:**
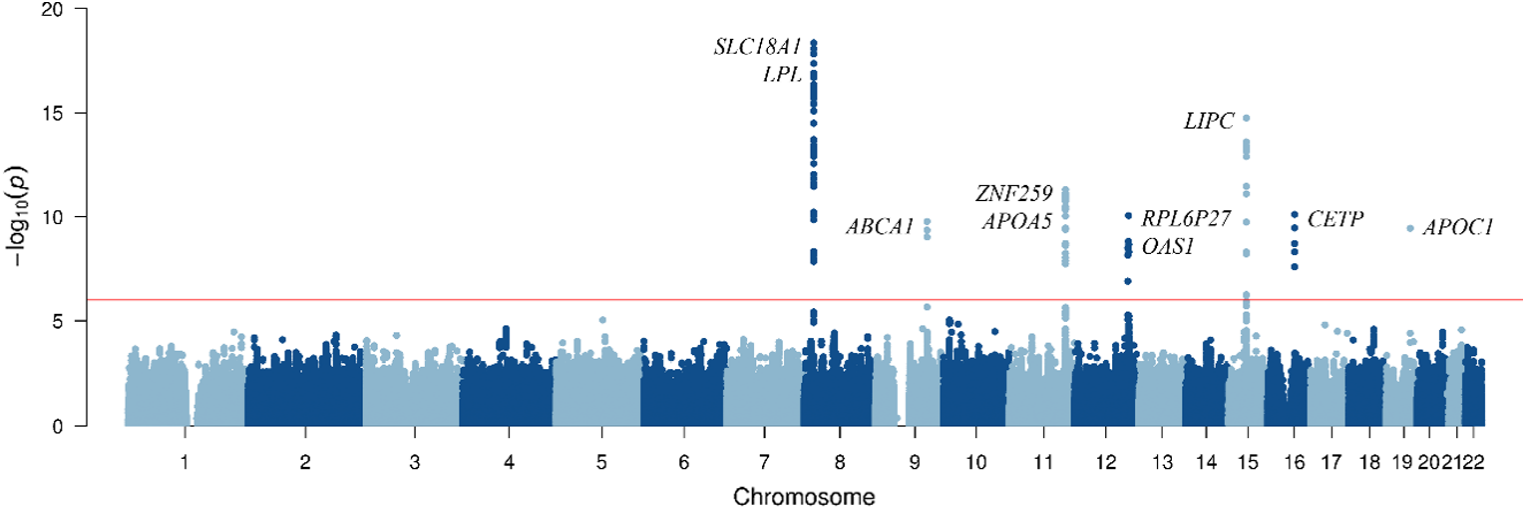
Manhattan plot of the results of genome‐wide association analysis of high‐density lipoprotein HDL cholesterol levels. The *x* axis shows the chromosomal position, and the *y* axis shows the −log_10_ *p*‐values using the trend test for SNPs distributed across the entire genome. The red line indicates the signals with *p* < 10^–6^ detected in the genome‐wide association study (GWAS). A total of 1,590,162 SNPs were used to generate the plot. SNP: single nucleotide polymorphism

Genome‐wide association study was performed to identify significant loci related to hypo‐HDL‐C. Among the 86,465 significant SNPs (*p* < .05), 165 were selected based on −log_10_ *p*‐value >6. We identified 18 SNPs after screening for crucial effects on HDL‐C levels (Table 3; Figure 2). These SNPs were distributed over seven chromosomes containing 13 candidate genes (Figure S1). Three SNPs were found on chromosome 8, from 19.9 Mb to 20.0 Mb, close to *LPL* and *SLC18A1*, while a single SNP was only significantly annotated on the adenosine triphosphate (ATP)‐binding cassette subfamily A member 1 (*ABCA1*) on chromosome 9. Five SNPs were located in the *BUD13* homolog, *ZPR1* zinc finger (*ZNF259*), and *APOA5* genes around 116.1 Mb to 116.2 Mb on chromosome 11, respectively. Among them, three SNPs were located on the same LD block (Figure S2). In the region of 111.3 Mb to 111.9 Mb on chromosome 12, three SNPs were located in the myosin light chain 2 (*MYL2*), ribosomal protein L6 pseudogene 27 (*RPL6P27*), and 2′‐5′‐oligoadenylate synthetase 1 (*OAS1*) genes. Another three SNPs were located in the aquaporin 9 (*AQP9*) and *LIPC* genes from 56.4 kb to 56.5 kb on chromosome 15. Two SNPs were commonly located in *CETP* on chromosome 16. The last significant SNP was annotated on the apolipoprotein C1 (*APOC1*) gene on chromosome 19. The rs6999158 SNP showed the most significant association with HDL levels (−log_10_ *p*‐value = 18.4) and was in the intergenic region near *SLC18A1*. The second most significant SNP, rs1011685, was located in *LPL*. rs16940212, rs2160669, and rs2075291 were located close to *LIPC*, *ZNF259*, and *APOA5*, respectively. The SNP with the highest absolute beta value (−2.6) was rs2075291 and had a significant effect on hypo‐HDL‐C (−log_10_ *p*‐value < 6). Based on the 18 SNPs with the strongest effect on hypo‐HDL‐C, we calculated GRS for further analysis.

**TABLE 3:** Genetic characteristics of the 18 SNPs causally associated with high‐density lipoprotein (HDL)‐cholesterol levels among Korean adults

Chr.	SNP	Gene	Position	Effected alleles	Other alleles	MA	MAF	Beta	S.E	Lower 95% CI	Upper 95% CI	‐log_10_ *p*‐value
8	rs271	*LPL*	19,857,982	A	G	A	0.2	1.0	0.2	0.7	1.4	8.3
8	rs1011685	*LPL*	19,875,049	T	C	T	0.1	1.9	0.2	1.4	2.3	16.9
8	rs6999158	*SLC18A1* ^a^	19,972,293	A	T	A	0.2	1.6	0.2	1.2	1.9	18.4
9	rs1883025	*ABCA1*	106,704,122	C	T	T	0.2	−1.1	0.2	−1.4	−0.7	9.8
11	rs180363	*BUD13* ^a^	116,103,099	C	T	C	0.2	1.1	0.2	0.8	1.5	8.0
11	rs180327	*BUD13*	116,128,869	T	C	C	0.4	−0.9	0.1	−1.2	−0.6	8.2
11	rs918144	*BUD13*	116,139,035	T	C	T	0.5	1.0	0.1	0.7	1.3	10.8
11	rs2160669	*ZNF259* ^a^	116,152,817	T	C	C	0.2	−1.2	0.2	−1.6	−0.9	11.3
11	rs2075291	*APOA5*	116,166,602	C	A	A	0.0	−2.6	0.4	−3.4	−1.9	11.1
12	rs2188380	*MYL2* ^a^	109,870,510	T	C	C	0.1	−1.3	0.2	−1.7	−0.9	8.5
12	rs11066280	*RPL6P27* ^a^	111,302,166	T	A	A	0.2	−1.2	0.2	−1.6	−0.9	10.1
12	rs11066453	*OAS1*	111,850,004	A	G	G	0.1	−1.3	0.2	−1.7	−0.9	8.7
15	rs11071371	*AQP9* ^a^	56,363,518	C	T	T	0.1	−1.1	0.2	−1.6	−0.7	6.3
15	rs16940212	*LIPC* ^a^	56,481,312	T	G	T	0.3	1.2	0.2	0.9	1.5	14.8
15	rs261332	*LIPC*	56,514,617	A	G	A	0.2	1.0	0.2	0.7	1.4	8.2
16	rs6499863	*CETP* ^a^	55,549,518	G	A	A	0.1	−1.3	0.2	−1.8	−0.9	7.6
16	rs12708980	*CETP*	55,569,880	T	G	G	0.1	−1.5	0.2	−2.0	−1.0	9.5
19	rs4420638	*APOC1*	50,114,786	A	G	G	0.1	−1.4	0.2	−1.9	−1.0	9.4

Note

Chr, chromosome; CI, confidence interval; MA, minor allele; MAF, minor allele frequency; SE, standard error; SNP, single nucleotide polymorphism.

Beta values were the effect size per additional effect allele.

^a^ SNP located in the intergenic region.

### 3.3 Interactive effects of LCDS and GRS on the prevalence of hypo‐HDL‐C

The ORs and 95% CIs of hypo‐HDL‐C across the interaction of LCDS tertiles and HDL levels are presented in Table 4. The hypo‐HDL‐C prevalence was analyzed based on changes in LCDS in each GRS level, and the lowest tertile (T1) in each GRS level was used as the reference group. In the GRS T3 group, subjects in the highest LCDS tertile had a significantly decreased hypo‐HDL‐C prevalence compared with those in LCDS T1 in both models 1 (*p for trend* = .0054) and 2 (*p for trend* = .0117). The subjects in the T2 and T3 LCDS groups in GRS T2 showed a borderline decrease, the hypo‐HDL‐C prevalence was not significant compared with that in LCDS T1 (*p for trend* = .0767, 0.0631 in models 1 and 2, respectively). The subjects in LCDS T3 in GRS T1 had significantly decreased hypo‐HDL‐C prevalence in model 1 (*p for trend* = 0.0264); however, the association was attenuated after adjustment in model 2 (*p for trend* = 0.1245), and no significant interaction term was observed.

**TABLE 4:** The odds ratios of hypo‐HDL‐C according to the LCDS tertiles stratified by the GRS among Korean adults

	LCDS T1	LCDS T2		LCDS T3		*p for trend* ^a^
	OR	OR	95% CI	OR	95% CI	
GRS T1	221/885 (25.0%)	234/969 (243, 25.1%)		189/917 (20.6%)		
Model 1	1.00 (ref.)	1.004	(0.813–1.240)	0.782	(0.626–0.976)	.0264
Model 2	1.00	1.026	(0.824–1.278)	0.814	(0.645–1.028)	.1245
GRS T2	315/921 (34.2%)	290/932 (290, 31.1%)		287/936 (30.7%)		
Model 1	1.00	0.873	(0.718–1.061)	0.839	(0.690–1.021)	.0767
Model 2	1.00	0.866	(0.706–1.062)	0.827	(0.672–1.017)	.0631
GRS T3	433/943 (45.9%)	381/890 (381, 42.8%)		363/921 (39.4%)		
Model 1	1.00	0.877	(0.729–1.056)	0.770	(0.640–0.926)	.0054
Model 2	1.00	0.873	(0.719–1.060)	0.769	(0.632–0.936)	.0117

Note

Model 1 was adjusted for sex and age. Model 2 was adjusted for sex, age, body mass index (BMI), energy intake, household income level, current drinking, smoking status, physical activity, and residence location.

Values are presented as case/total (%). The *p*‐values for the interaction were 0.6599 and 0.6922 for model 1 and model 2, respectively.

Abbreviations: Hypo‐HDL‐C: hypo‐HDL‐cholesterolemia, LCDS: low‐carbohydrate diet score, GRS: genetic risk score, T: tertile, KoGES: Korean Genome and Epidemiology Study, OR: odds ratio, CI: confidence interval, ref.: reference data of odds ratios of hypo‐HDL‐C analysis between the groups.

^a^ *p for trend* was linear trends across categories of LCDS tested using the median value for each category as an ordinal variable. The *p*‐value for the interaction was 0.0928.

**FIGURE 3:**
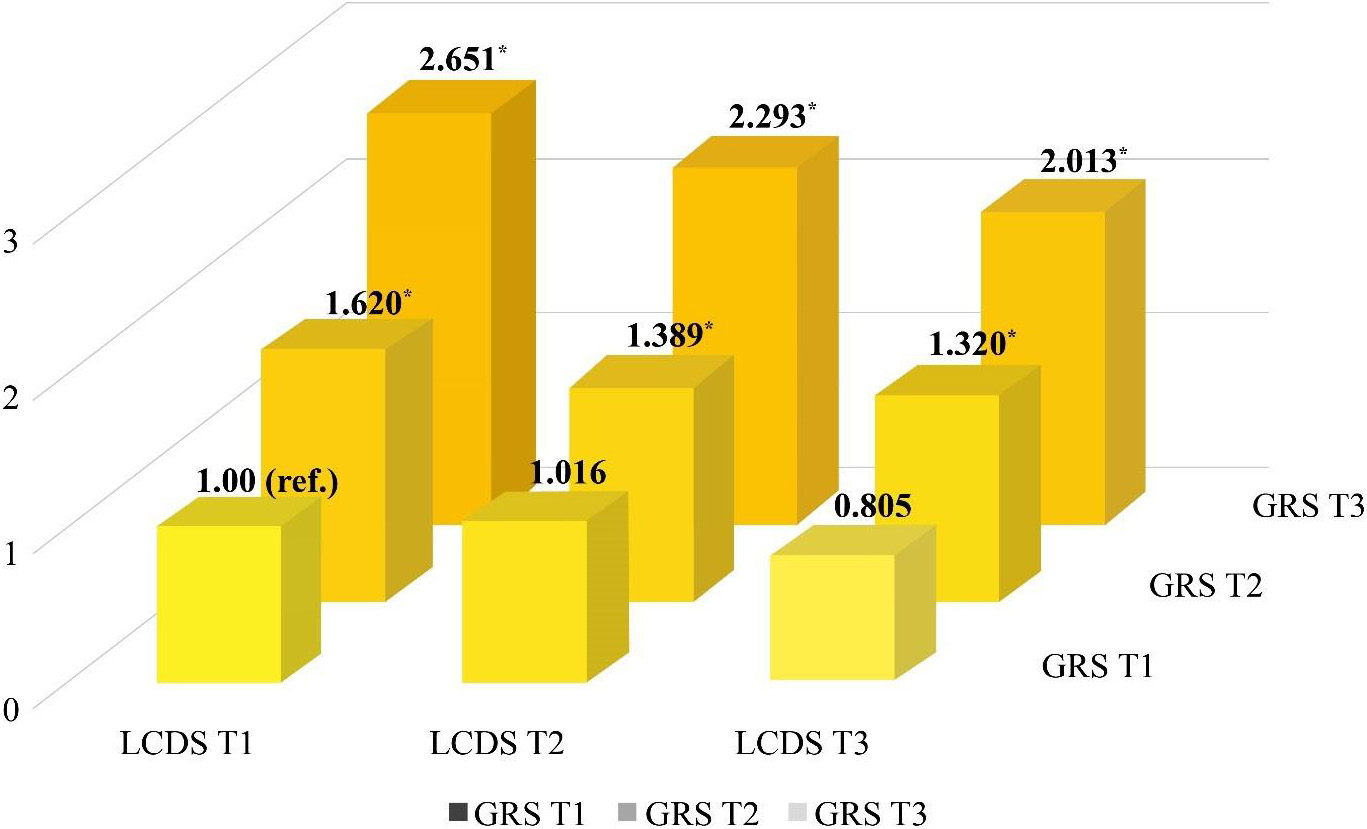
The odds ratios (ORs) (95% confidence intervals (CIs)) of hypo‐HDL‐C, according to the joint categories LCDS and GRS, among the Korean population in the Ansan and Ansung cohort of the KoGES. Hypo‐HDL‐C: hypo‐HDL‐cholesterolemia, LCDS: low‐carbohydrate diet score, GRS: genetic risk score, T: tertile, KoGES: Korean Genome and Epidemiology Study. *p‐value < .05

In the joint effect model with low LCDS and low GRS as a reference, the lowest prevalence risk for borderline hypo‐HDL‐C was observed in individuals with high (T3) LCDS and low (T1) GRS (*p for trend* < .0001). The highest prevalence risk was observed in individuals with low LCDS and high GRS (*p for trend* < .0001) (Table 5 and Figure 3). In addition, at any level of GRS, a higher LCDS decreased the hypo‐HDL‐C prevalence, and at any level of LCDS, a higher GRS increased the hypo‐HDL‐C prevalence.

**TABLE 5:** The odds ratios of hypo‐HDL‐C according to the joint categories LCDS and the GRS among Korean adults

	LCDS T1		LCDS T2		LCDS T3		*p for trend* ^a^
	OR	95% CI	OR	95% CI	OR	95% CI	
GRS T1	221/885 (25.0%)		234/969 (243, 25.1%)		189/917 (20.6%)		.1245
	1.00 (ref.)		1.016	(0.817–1.265)	0.805	(0.639–1.014)	
GRS T2	315/921 (34.2%)		290/932 (290, 31.1%)		287/936 (30.7%)		.0631
	1.620	(1.311–2.001)	1.389	(1.120–1.721)	1.320	(1.062–1.640)	
GRS T3	433/943 (45.9%)		381/890 (381, 42.8%)		363/921 (39.4%)		.0117
	2.651	(2.156–3.260)	2.293	(1.857–2.832)	2.013	(1.627–2.489)	
p for trend^*^	<.0001		<.0001		<.0001		

Note

Adjusted variables were sex, age, body mass index (BMI), energy intake, household income level, current drinking, smoking status, and physical activity.

Values are shown as case/total (%).

Abbreviations: CI, confidence interval; GRS, genetic risk score; Hypo‐HDL‐C, hypo‐HDL‐cholesterolemia; KoGES, Korean Genome and Epidemiology Study; LCDS, low‐carbohydrate diet score; OR, odds ratio; ref., reference data of odds ratios of hypo‐HDL‐C analysis between the groups; T, tertile.

^a^ *p for trend* is for each trend by GRS in LCDS, and LCDS in GRS.

## 4 DISCUSSION

We examined the interactions between LCDS based on the energy percentage of carbohydrate, protein, and fat intake and the GRS using 18 significant SNPs derived from the GWAS for HDL cholesterol. The LCDS not only indicated low‐carbohydrate content but also high protein and fat content, which could affect HDL cholesterol positively through the action of fats such as PUFA. A higher LCDS was associated with a decreased prevalence of hypo‐HDL‐C among subjects with a higher GRS but not in those with lower GRS. The lowest risk of hypo‐HDL‐C prevalence was observed in individuals with both low GRS for low HDL cholesterol level and high LCDS. However, no clear interaction term was observed between GRS and LCDS. These results suggest that a low‐carbohydrate diet might attenuate genetic influences on hypo‐HDL‐C.

The results showed that the risk of hypo‐HDL‐C significantly decreases as the LCDS increases among Korean adults, which is consistent with the findings in previous studies (Kim et al., 2019; Park et al., 2010). Consistent with the findings in other studies, a high LCDS increased HDL cholesterol levels (Brehm et al., 2003; Nordmann et al., 2006; Yancy et al., 2004), possibly through a mechanism underlying the effects of dietary macronutrient composition. A high LCDS diet derives its greatest proportion of energy from fat rather than from carbohydrate, which increases the HDL cholesterol level (Bazzano et al., 2014). Furthermore, the intake of lauric acid‐rich fats increases HDL cholesterol levels (Mensink et al., 2003).

Although hypo‐HDL‐C was significantly decreased by a high LCDS and low GRS, LCDS did not show a significant decrease in the prevalence of hypo‐HDL‐C in the highest GRS group, indicating that genetic factors also moderate the levels of HDL cholesterol. Similar results have shown that the highest risk of hypo‐HDL‐C occurred in subjects with presumably decreased activities of two proteins due to an interaction between *LPL* and *CETP* polymorphisms (Corsetti et al., 2011). A high level of TG‐rich lipoproteins caused *CETP*‐induced transfer of cholesteryl ester from HDL to TG‐rich lipoproteins in exchange for TG resulting in the formation of small HDL particles that are catabolized faster than large HDL particles, leading to lower levels of HDL cholesterol (Welty, 2013).

The effects of three candidate genes, including *ABCA1*, *APOA1*, and *LCAT*, on low HDL cholesterol levels have been investigated in Dallas residents and Canadians (Cohen et al., 2004). Among people with low HDL cholesterol levels, 10%–15% of them had ABCA1 mutations (Frikke‐Schmidt et al., 2004). These SNPs (*APOA‐I*, *ABCA1*, and *SR‐BI*) disrupt the pathway of HDL biogenesis and may lead to dyslipidemia and atherosclerosis in mice. The SNP in *BUD13*, a main SNP in our study, was similarly highly associated with metabolic syndrome and hyperlipidemia in Asian countries, such as China and Taiwan (Aung et al., 2014; Lin et al., 2016). However, in the populations recruited in Minnesota, different genetic SNPs, such as those in the *KCTD_10_* and *MMAB* genes, were found to possibly contribute to modulating the HDL cholesterol level in individuals with high‐carbohydrate intakes (Junyent et al., 2009). This suggests that Asians and Americans have different genetic risks.

For GRS‐related HDL, the Framingham Heart Study evaluated the GRS for lipid levels using genome‐wide markers based on their study. Lipid levels, including HDL cholesterol, were more highly associated with weighted than with unweighted GRS variables (Piccolo et al., 2009). CVD prevalence among participants with familial hypercholesterolemia indicated that the GRS based on related SNPs could modify disease phenotype, facilitating a personalized therapy approach (Paquette et al., 2017).

Large‐scale analysis of GWAS has advanced our understanding of the role of genetic variation in complex human diseases, such as diabetes (Barrett et al., 2009; Frayling et al., 2007; Onengut‐Gumuscu et al., 2015). GWAS has provided new insights into disease mechanisms and associations with dietary factors (Brunkwall et al., 2016). Despite reductions in the cost of genotyping, a direct effect on clinical care for individuals with hypo‐HDL‐C based on dietary factors and their interactions with genetic predisposition has not yet been developed. Thus, these results provide important insights into the interactive effects of GRS and LCDS on hypo‐HDL‐C, which may contribute to establishing personalized diet plans based on an individual's genetic predisposition in the future.

This study has few limitations. First, this study was designed as a cross‐sectional study, which cannot analyze the incidence of hypo‐HDL‐C. Second, the Ansan and Ansung study does not represent the whole Korean population. Lastly, even though LCDS T3 was the highest score, the mean %E of carbohydrates was 64.6% in the T3 of LCDS, which means that the LCDS of this study cannot be generalized for other populations. Thus, to examine our results further, studies on populations with lower carbohydrate intake (<45%E) are needed to examine the interactive effect of low‐carbohydrate diet and GRS on hypo‐HDL‐C.

Despite the limitations, our study has the following key strengths. The results of the present study serve as a foundation for evaluating the joint effect between dietary intake and genetic factors in hypo‐HDL‐C using GWAS. Previous studies were designed for the separate association analysis of dietary or genetic factors or of the interactive effects of each dietary factor with each SNP on hypo‐HDL‐C. We calculated the GRS as an indicator of an individual's HDL cholesterol levels, not associated with the TG and LDL cholesterol levels. This study may contribute to establishing personalized diet plans for patients trying to control hypo‐HDL‐C based on genetic risk and to the improvement of public health, especially in the Korean population that has a high carbohydrate intake ratio. Diets might facilitate the prevention of hypo‐HDL‐C, especially those with SNPs associated with low HDL cholesterol levels. Individuals with high GRS are advised to implement low‐carbohydrate and high‐fat diets to prevent hypo‐HDL‐C. Although the present study does not account for the entire Korean population, the general characteristics of the Ansan and Ansung populations and in the Korean National Health and Nutrition Examination Surveys represent those of the entire Korean population. Further studies are required to clarify the mechanism underlying the interactive effects of LCDS and GRS on hypo‐HDL‐C. Furthermore, larger cohort studies, such as residential extended studies, are required to verify that the study results adequately account for the entire Korean population.

## CONFLICT OF INTEREST

The authors declare that they have no competing interests.

## ETHICAL APPROVAL

The protocol of the current study was approved by the Institutional Review Board (IRB) of Chung‐Ang University (IRB no. 1041078–201,908‐HRBR‐239–01).

## CONSENT TO PARTICIPATE

Written informed consent forms were signed by all participants.

## CONSENT FOR PUBLICATION

Not applicable.

## Supporting information

Supplementary Material

## Data Availability

The datasets used and/or analyzed during the current study are owned by a third‐party organization (The Korean Genome and Epidemiology Study‐Ansan and Ansung Study, KoGES; 4851–302). These data are available by online sharing service under the permission of the division of epidemiology and health index in the Korea Centers for Disease Control and Prevention (KCDC). More details in English are available on the following website: URL: http://www.nih.go.kr/contents.es?mid=a50401010400#1.
