# Supplementary material for: Interactive effects of the low‐carbohydrate diet score and genetic risk score on Hypo‐HDL‐cholesterolemia among Korean adults: A cross‐sectional analysis from the Ansan and Ansung Study of the Korean Genome and Epidemiology Study
Source: Food Sci Nutr. 2022 Apr 29;10(9):3106–16. doi: 10.1002/fsn3.2909 (PMC9469851; doi:10.1002/fsn3.2909)
Supplement: Supplementary file 1 — Supplementary Material [file FSN3-10-3106-s001.docx]

Supplementary Information

Interactive Effects of the Low-carbohydrate Diet Score and Genetic Risk Score on Hypo-HDL-Cholesterolemia Among Korean Adults: A Cross-sectional Analysis from the Ansan and Ansung Study of the Korean Genome and Epidemiology Study

SoHyun Park, Min-Jae Jang, Min Young Park, Jun-Mo Kim^*^, Sangah Shin^*^

^*^Corresponding authors:

Sangah Shin, ORCID: <https://orcid.org/0000-0003-0094-1014>

Department of Food and Nutrition, Chung-Ang University, 4726, Seodong-daero, Daedeokmyeon, Anseong-si, Gyeonggi-do 17546, Republic of Korea

Tel: +82-31-670-3259, Fax: +82-31-675-1381, E-mail: [ivory8320@cau.ac.kr](mailto:ivory8320@cau.ac.kr)

Jun-Mo Kim, ORCID: http://orcid.org/0000-0002-6934-398X

Department of Animal Science and Technology, Chung-Ang University, 4726, Seodong-daero, Daedeokmyeon, Anseong-si, Gyeonggi-do 17546, Republic of Korea

Tel: +82-31-670-3263, Fax: +82-31-675-1381, E-mail: [junmokim@cau.ac.kr](mailto:junmokim@cau.ac.kr)

**Supplementary Table 1.** Nutritional intake according to LCDS tertile among the Korean population in the Ansan and Ansung study of the KoGES.

|  | LCDS T1 | LCDS T2 | LCDS T3 | *p-value** |
| --- | --- | --- | --- | --- |
| N | 2749 | 2791 | 2774 |  |
| Calcium (mg) | 332.1 ± 178.5 | 473.6 ± 211.9 | 617.5 ± 277.7 | <0.0001 |
| Phosphorus (mg) | 811.3 ± 293.8 | 1011.0 ± 302.9 | 1228.9 ± 397.3 | <0.0001 |
| Iron (mg) | 8.5 ± 4.0 | 10.9 ± 4.2 | 13.0 ± 5.0 | <0.0001 |
| Potassium (mg) | 2034.1 ± 999.9 | 2548.8 ± 1017.4 | 2981.3 ± 1114.7 | <0.0001 |
| Sodium (mg) | 2683.3 ± 1456.2 | 3178.7 ± 1462.9 | 3660.8 ± 1685.2 | <0.0001 |
| Zinc (µg) | 7.1 ± 2.8 | 8.5 ± 3.2 | 10.6 ± 5.3 | <0.0001 |
| Folate (µg) | 201.9 ± 110.7 | 250.6 ± 116.4 | 284.8 ± 126.8 | <0.0001 |
| Carotene (µg) | 2127.7 ± 1941.9 | 2772.8 ± 2225.3 | 3333.0 ± 2547 | <0.0001 |
| Fiber (g) | 6.2 ± 3.3 | 7.2 ± 3.2 | 7.6 ± 3.2 | <0.0001 |
| Vitamins |  |  |  |  |
| Vitamin A (R.E.) | 396.5 ± 321.3 | 534.7 ± 365.3 | 673 ± 424.7 | <0.0001 |
| Retinol (µg) | 34.2 ± 30.5 | 63.8 ± 44.1 | 105.0 ± 74.5 | <0.0001 |
| Vitamin B1 (mg) | 1.0 ± 0.4 | 1.2 ± 0.4 | 1.5 ± 0.6 | <0.0001 |
| Vitamin B2 (mg) | 0.7 ± 0.3 | 1.0 ± 0.4 | 1.3 ± 0.5 | <0.0001 |
| Niacin (mg) | 12.3 ± 4.5 | 15.1 ± 4.9 | 19.1 ± 7.0 | <0.0001 |
| Vitamin B6 (mg) | 1.5 ± 0.6 | 1.8 ± 0.6 | 2.1 ± 0.8 | <0.0001 |
| Vitamin C (mg) | 113.4 ± 97.6 | 129.9 ± 92.0 | 138.5 ± 85.4 | <0.0001 |
| Vitamin E (mg) | 7.2 ± 3.8 | 9.4 ± 4.5 | 11.3 ± 5.1 | <0.0001 |

LCDS: low carbohydrate diet score, T: tertile, KoGES: Korean Genome and Epidemiology Study.

Values are shown as the mean ± standard deviation. ^*^*p*-*values* were obtained from a generalized linear model for continuous variables.


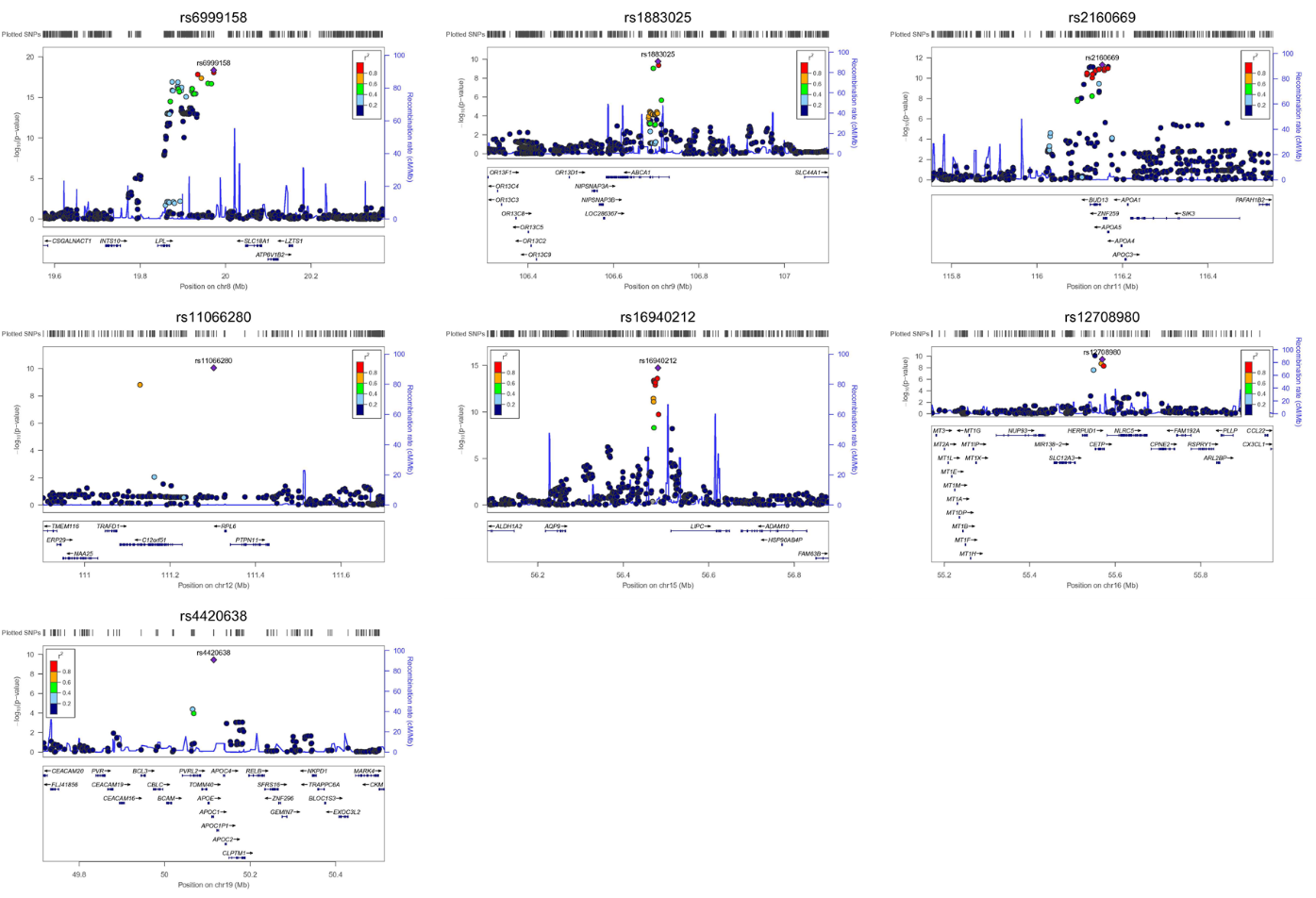


**Supplementary Fig. 1** Regional association plots for significant hypo HDL cholesterolemia loci. At the top, the positions of SNPs are shown, and in the middle, the regional association results from the genome-wide association study are shown. The test –log_10_ *P* values are shown for SNPs distributed in an 0.8-MB genomic region centered on the most strongly associated signal, which is depicted as a purple diamond. The genetic information was from the Human Genome build hg18, and the LD structure was based on the HapMap Phase Ⅱ JPT + CHB


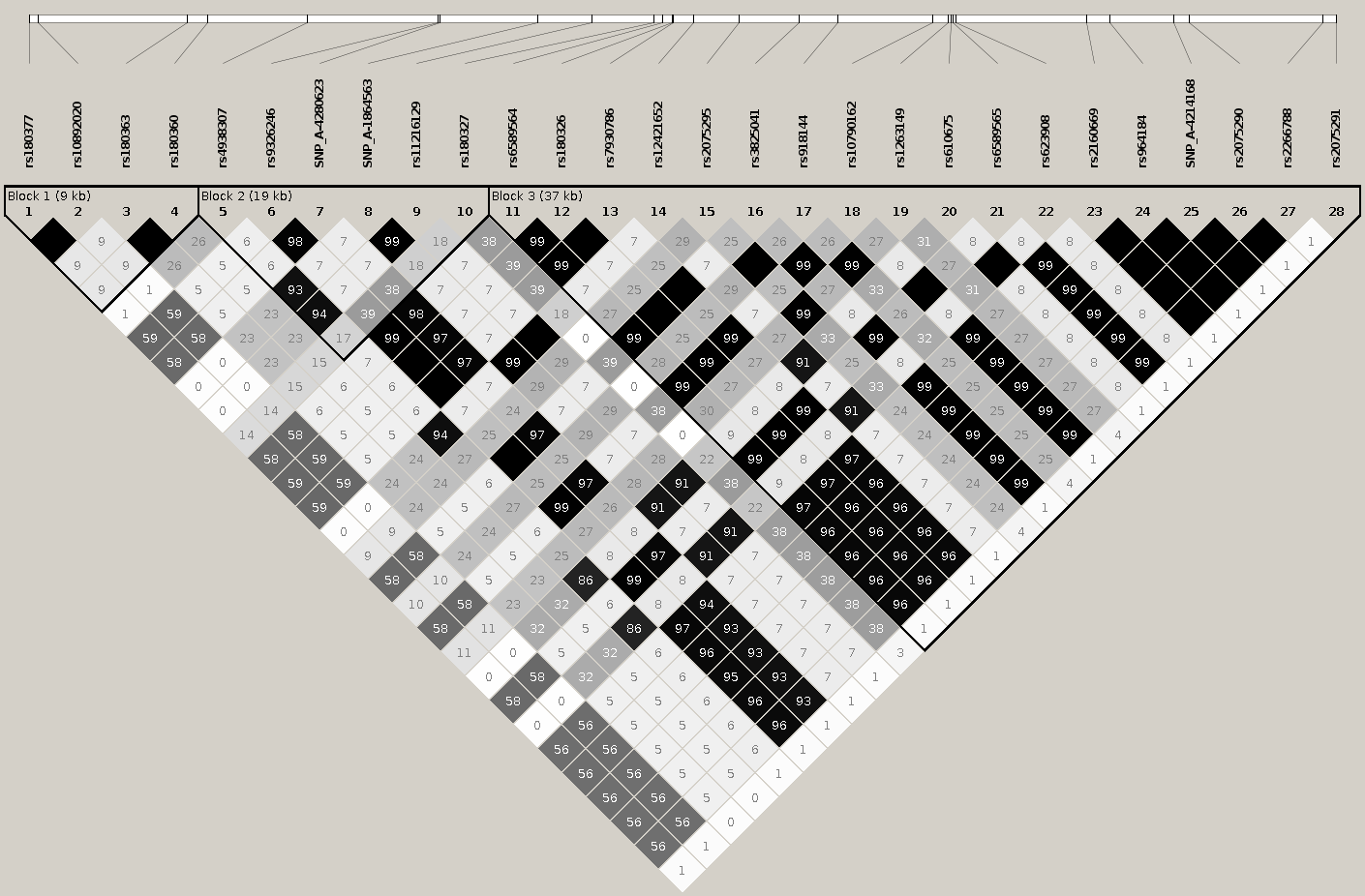


**Supplementary Fig. 2** Linkage disequilibrium (LD) block of SNPs located in chromosome 11. LD block was generated by Haploview 4.2 software. The LD measure shown is *r^2^* and the block definition is defined by the Four gamete rule. The significant three SNPs are indicated in bold squares
